# Evaluating Molecular Properties Involved in Transport of Small Molecules in Stratum Corneum: A Quantitative Structure-Activity Relationship for Skin Permeability

**DOI:** 10.3390/molecules23040911

**Published:** 2018-04-15

**Authors:** Chen-Peng Chen, Chan-Cheng Chen, Chia-Wen Huang, Yen-Ching Chang

**Affiliations:** 1 Department of Occupational Safety and Health, College of Public Health, China Medical University, No. 91 Hsueh-Shih Road, Taichung 40402, Taiwan; chencp@mail.cmu.edu.tw (C.-P.C.); u100014014@cmu.edu.tw (C.-W.H.); yen071013@gmail.com (Y.-C.C.); 2 Department of Safety, Health and Environmental Engineering, National Kaohsiung University of Science and Technology, No.1, University Road, Yanchao District, Kaohsiung City 824, Taiwan

**Keywords:** quantitative structure-activity relationship, skin permeability, molecular weight, octanol-water partition coefficient, antineoplastic property, application domain

## Abstract

The skin permeability (*Kp*) defines the rate of a chemical penetrating across the stratum corneum. This value is widely used to quantitatively describe the transport of molecules in the outermost layer of epidermal skin and indicate the significance of skin absorption. This study defined a *Kp* quantitative structure-activity relationship (QSAR) based on 106 chemical substances of *Kp* measured using human skin and interpreted the molecular interactions underlying transport behavior of small molecules in the stratum corneum. The *Kp* QSAR developed in this study identified four molecular descriptors that described the molecular cyclicity in the molecule reflecting local geometrical environments, topological distances between pairs of oxygen and chlorine atoms, lipophilicity, and similarity to antineoplastics in molecular properties. This *Kp* QSAR considered the octanol-water partition coefficient to be a direct influence on transdermal movement of molecules. Moreover, the *Kp* QSAR identified a sub-domain of molecular properties initially defined to describe the antineoplastic resemblance of a compound as a significant factor in affecting transdermal permeation of solutes. This finding suggests that the influence of molecular size on the chemical’s skin-permeating capability should be interpreted with other relevant physicochemical properties rather than being represented by molecular weight alone.

## 1. Introduction

### 1.1. Identification of Transdermal Penetration for Manmade and Natural Chemicals

The exposure of the skin to manmade and naturally derived chemicals is an issue of rising concern, particularly in the workplace, where dermal absorption represents a prominent route by which significant uptake of hazardous chemicals may occur [1,2]. To reduce the risk of occupational skin exposure, authoritative agencies and organizations worldwide have adopted skin notations (SNs) as a part of their occupational exposure limits in the management of skin exposure hazards [1,2,3]. The SNs are a qualitative, dichotomous indicator that alerts workers of the presence of chemicals capable of permeating through human skin at a significant level and consequently provoking systemic toxicity. However, the SNs have not been used effectively in the management of occupational skin exposure, as robust data reporting systemic/target organ toxicity as a direct result of skin absorption are required to support the hazard identification of toxic compounds [2,4]. The lack of sufficient data from biological tests in vivo and in vitro to demonstrate potential dermal penetration and absorption of toxic compounds has been a major factor impeding the development of quantitative standards in the management of occupational skin exposure.

The significance of skin absorption for a target compound is conventionally evaluated by determining the skin permeability (*Kp*), or the skin permeation coefficient, of the compound in the stratum corneum. Quantitatively, the *Kp* describes the rate of chemical permeation through the outermost layer of the epidermal skin [4]. This value may be determined in vivo, but it is more frequently determined in vitro following the protocols developed by, e.g., the Organisation for Economic Cooperation and Development (OECD) [5,6]. In the in vitro method, human or animal cadaver skin is used, and the permeation of typically non-radio-labeled compound through the skin is monitored using the static or flow-through diffusion-cell technique. The *Kp* presents a measure by which the potential of biological uptake via the skin for a compound can be quantified, and it has been relied on as a significant source of data to support dermal hazard evaluation [2]. For example, the US Environmental Protection Agency (USEPA) at the request of the US Occupational Safety and Health Administration (OSHA), issued standardized protocols on in vitro dermal absorption rate testing for the evaluation of industrial chemicals of interest to the OSHA [7]. The US National Institute for Occupational Safety and Health (NIOSH), in its new strategy for the assignment of SN, also recommended the use of *Kp* as a criterion in the decision-making process [8]. However, the biological testing and derivation of *Kp* was subject to constraints inherent in the experimental techniques, e.g., the duration of the test, the origin and thickness of the skin used in the test, the formulation of the chemical employed as test material, the dosing scheme, and the potential use of a vehicle in the topical application of chemicals for enhancement of transdermal penetration. As a consequence, conventional *Kp* tests frequently generated data of a quality insufficient to support adequate interpretation of dermal exposure risk.

### 1.2. Predictive Modeling of Skin Permeation

In recent years, the quantitative structure-activity relationship (QSAR) has received great attention as a strategy in the assessment of skin exposure risk, and a variety of QSARs have been attempted to provide a viable means of *Kp* prediction [9]. Regulatory agencies worldwide have stepped up the use of predictive algorithms in the identification of skin exposure hazards when the data reported from biological testing alone have been insufficient to support an adequate assessment. For example, the American Conference of Governmental Industrial Hygienists, in its Threshold Limit Value Documentation [3], recommended: when a chemical was evaluated for a skin designation, the “extrapolations of systemic effects from other routes of exposure suggest dermal absorption may be important in the expressed toxicity” should be considered, in addition to the reports of acute/repeated-dose toxicity (e.g., dermal lethal dose 50%), and any indications of potential dermal absorption (e.g., logarithmic octanol-water partition coefficient, log *K_OW_*). In practice, the extrapolation of systemic toxicity from other routes of exposure has been realized as a comparison of the chemical’s dose absorbed via the skin to the level absorbed by route of inhalation during the same period of exposure [4]. The European Centre for Ecotoxicology and Toxicology of Chemicals (ECETOC), in its strategy for the assignment of SNs [10], adopted a similar scheme in the determination of dermal absorption potential, in which the level of the predicted skin absorption for the scrutinized compound was compared to the level known or estimated to elicit specific systemic effect(s). The ECETOC further recommended the use of inference from physicochemical properties or structure-activity relationships to facilitate assessment of dermal absorption potential. In response to the request from the OSHA for data on the dermal absorption rate of toxic industrial chemical, the US Toxic Substances Control Act Interagency Testing Committee proposed a method to estimate the skin absorption time based on the inhalational-to-dermal extrapolation of acceptable biological uptake for chemical capable of penetrating across the skin and provoking systemic toxicity [11]. This algorithm was later transformed to serve as a criterion in the renovated NIOSH strategy for assignment of NIOSH SNs [8]. A theme in common among these mechanisms of route-to-route extrapolation for a skin exposure hazard was the use of *Kp* in deriving at a threshold for the identification of significant skin absorption. The *Kp*-predicting QSARs, when adequately validated with data from finite-dose dermal absorption testing, provided a viable approach for meeting the data need in the dermal risk assessment [12], and thus their regulatory application is expected to continue and perhaps be expanded to facilitate the process of decision-making.

### 1.3. Quantitative Structure-Activity Relationship for Skin Permeation Estimation

The *Kp* QSARs reported in the literature in majority are mechanistically or empirically based (correlation-based). Lian et al. [13], in their review, indicated that these two types of QSARs differed primarily in their selection of molecular descriptors when describing the behavior of compounds traversing across the viable epidermis. Table 1 presents a list of *Kp* QSARs to demonstrate the evolution of *Kp* QSAR and the selection of molecular descriptors. In the list, the QSAR developed by Mitragotri [14] has been included to represent the mechanistically based QSAR.

In the early days of *Kp* QSAR development, the molecular descriptors considered to be influential to the transdermal transport of chemical and thus included in the model were typically those of a measurable physicochemical property, e.g., molecular weight (MW), melting point (MP), and log *K_OW_*. The MW and log *K_OW_* were often the key—and in some cases the only—descriptors in the correlation-based QSARs developed in this era [22,23], e.g., the Potts and Guy QSAR initially developed in 1992 [15]. The prediction of *Kp* in the QSAR using measurable physicochemical properties, however, hindered the appreciation of molecular characteristics that might not be readily measured but interacted to exert influence on the transepidermal transport of molecules. For example, in their validation of five different *Kp* QSARs consisting of only MW and log *K_OW_* as descriptors, Wilschut et al. [22] suggested that, considering the dense distribution of electrons, for the compounds of an aromatic structure the influence of molecular size to *Kp* might be better represented by molecular volume (MV) than by MW. As a result of an emphasis on the physicochemical properties, the early *Kp* QSARs were, in general, of a predictive power insufficient to quantitatively describe the transport behavior of molecules in the stratum corneum. In recent years, the descriptors presented in the *Kp* QSARs shifted from those characteristic of physicochemical properties to those of relevance to the arrangement of atomic and electrical occupation in the molecular space (e.g., MV) and the electronic distribution in that space (e.g., hydrogen bonding). Aiming to better delineate the processes underlying the movement of molecules in the epidermal skin, the shift in the selection of molecular descriptors also provided an opportunity to investigate any potential interactions among molecular characteristics that could not be explained by measurable physicochemical properties. For example, the QSAR that Potts and Guy established in 1992 [15] was revised and published in 1995 to predict *Kp* based on the MV and the acidity/basicity of the solute hydrogen bond [18].

As the QSAR methodology continues to improve, computer programs are available nowadays to better envision how different molecular characteristics interact to influence the targeted effect (activity). For example, the software Dragon^®^ for molecular descriptor calculation and analysis [24] in its version 5.5 provides calculation for over three thousand descriptors. Many of these descriptors, such as the geometrical descriptors and topological descriptors, were less thoroughly attempted in the previous *Kp* QSAR. The advancement in the modeling technique offers an opportunity to re-define the *Kp* QSAR and to explore the molecular characteristics involved in driving the transdermal transport of small molecules.

### 1.4. Study Goals

The study reported in this article defined a *Kp* QSAR consisting of four molecular descriptors, as developed using *Kp* of 106 compounds measured for human skin. This *Kp* QSAR characterized, at a molecular level, the mechanisms involved in transdermal permeation of small-molecule solutes. This QSAR also identified a sub-domain of molecular properties that described the resemblance of a compound to antineoplastics as a significant factor affecting the transdermal transport of solutes. A comparison was made of the *Kp*-predicting power between the model developed in this study and those presented in Table 1. The results of the comparison suggested that the current model was of a capacity sufficient to serve as an alternative source of *Kp* data in support of dermal hazard identification.

## 2. Results and Discussion

### 2.1. Development of Quantitative Structure-Activity Relationship

In this study, the stepwise regression algorithm selected regressor variables from a total of 1530 candidate molecular descriptors for inclusion in the multiple linear regression (MLR)-based *Kp* QSAR. As the pool of candidates to select the descriptors from was significant, the *p* values assigned in the hypotheses for the addition of regressors to the model and for the removal from the model were crucial to the number of descriptors to include in the final QSAR. To control the number of molecular descriptors in the QSAR for reasons of model accessibility and mathematical maneuverability, the strategy applied in the descriptor selection in this study was to combine a higher probability of removing descriptors and a lower probability of adding descriptors. In this study, the *p* value for a regressor leaving the model was set at 0.1, and its counterpart for a regressor to enter was 5 × 10^–^^6^. Once the effective molecular descriptors had been determined, the parameters in the MLR model were calculated based on a training set of 85 compounds. Afterwards, a validation dataset of 21 compounds was used to validate the predictive capability of the developed MLR model. These processes of model training and validation generated a final MLR model of four molecular descriptors:

(1) $$\begin{array}{cc} \log\;Kp= & -3.0943(\pm 0.0923)-0.0067(\pm 0.0006)D/\mathrm{Dr}10-0.0496(\pm 0.0103)T(O..\mathrm{Cl})\\ & +0.6840(\pm 0.0407)\;\mathrm{ALOGP}-1.5709\;(\pm 0.1175)\;\mathrm{Neoplastic}-80 \end{array}$$

The log *Kp* QSAR identified four molecular descriptors as statistically significant molecular characteristics capable of affecting the transport behaviors of solutes in the stratum corneum (Table 2). In Equation (1), the figures in the parentheses before each descriptor were the standard errors estimated for the corresponding parameters. The analysis of variance was performed to evaluate the fitting ability of the model to the training dataset (Table 3); a very low *p* value (*F* statistic = 121.3; *p* < 0.001) was observed, indicating a significant fit of the model to the training compounds.

### 2.2. Performance of Quantitative Structure-Activity Relationship

**Figure 1:**
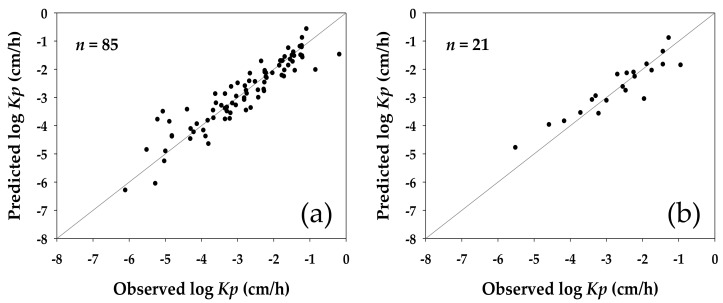
Distribution of logarithmic skin permeability predicted by quantitative structure-activity relationship developed in this study (predicted log *Kp*) against values experimentally observed (observed log *Kp*) for (**a**) 85 compounds in the model training dataset, and for (**b**) 21 compounds in the model validation dataset. Diagonal solid lines in the graphs represent where the predicted log *Kp* would equal the observed log *Kp* for a target compound.

To evaluate the performance of the developed *Kp* QSAR, the log values of experimental *Kp* for the compounds included in the datasets were compared to those predicted by the model. Figure 1 shows the relative distribution of the log *Kp* predicted by the model against their counterparts observed in the original experiments for the compounds in the training and validation sets; and Table 4 summarizes the fitting ability and predictive capability of the developed model in statistical terms. As the analysis demonstrated, there were no significant outliers found in either case.

A rule of thumb commonly adopted in the development of QSAR for practical application is: the difference between *R*^2^ and *Q*^2^ must not be too large and preferably not exceeding 0.2–0.3. In addition, a value of *Q*^2^ greater than 0.5 is regarded as an indication of good performance, and a value greater than 0.9 as an indication of excellent performance [25]. As shown in Table 4, the fitting ability, as demonstrated in *R*^2^ for the developed *Kp* QSAR, was 0.858; and the predictive capability, as in *Q*^2^, was 0.839. The difference between *R*^2^ and *Q*^2^ of the proposed model was 0.019. These results suggested that the developed QSAR was of an adequate predictive power for the estimation of log *Kp*. Moreover, the level of error observed in the predicted value of *Kp* was of a reasonable level, considering the variation inherent in the experimental determination of *Kp*.

**Figure 2:**
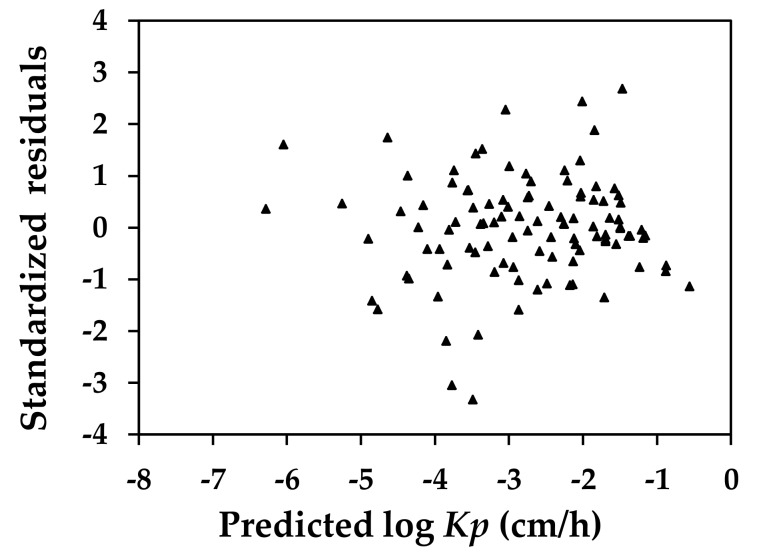
Scatter plot of standardized residuals versus logarithmic values of skin permeation coefficient (*Kp*) predicted using *Kp* quantitative structure-activity relationship (predicted log *Kp*) developed in this study for compounds included in in training and validation datasets.

Figure 2 shows the distribution for the standardized residuals of the prediction versus the predicted log *Kp* values. The result of the homoscedasticity test suggested a consistent performance of the current QSAR in predicting the *Kp* for compounds of varying molecular characteristics. In the figure, the residuals for *N*-nitrosodiethanolamine and atropine were ‒3.05 and ‒3.33, respectively. While these numbers were slightly more than three standardized deviations, these two compounds were not considered to be heterogeneous, given the significant variance that might be present in the processes by which these values were generated. As we introduced, when the rate of chemical permeation in the epidermal skin was experimentally determined, the procedures employed could differ significantly. As a result it was not uncommon to observe a variation of a magnitude of two orders in the *Kp* values determined for the same compound when different procedures were applied in the experiment. van de Sandt et al. [26] examined the intra- and inter-laboratory variation in the results of in vitro percutaneous absorption tests conducted among 10 European laboratories using human donor skin for three compounds of varying physicochemical properties: benzoic acid, caffeine, and testosterone. The examination reported a coefficient of variation of 6.3–52.5%, 12.0–91.4%, and 6.3–111.0% for benzoic acid, caffeine, and testosterone, respectively. The log *Kp* of *N*-nitrosodiethanolamine used in developing the current QSAR was of a low value, –5.22 (the *Kp* was approximately 6.02 × 10^–6^ cm/h) [27]. This value was comparable to the *Kp* observed for *N*-nitrosodiethanolamine in the experiment where water was used as a vehicle [28]; however, it was over 180 folds less than the level (1.1–4.1 × 10^–3^ cm/h) determined when neat (undiluted) isopropyl myristate, a widely used lipoidal compound, was used as the vehicle for topical administration [28,29,30].

### 2.3. Comparison of Current Model with Quantitative Structure-Activity Relationships Reported in Literature

In our study, we limited our introduction to and comparison with the QSARs of *Kp* prediction only to those that were developed using datasets comparable to the data included in this study. As these models were representative of the *Kp* QSARs developed in a different era, and many are still in wide application, this approach provided an opportunity for us via a comparison among the models to observe how the interpretation of molecular characteristics governing skin permeation of compounds changed in response to the improvement in QSAR molecular representation. Many of the models being compared in this study remain benchmarks in *Kp* QSAR development [31,32,33], despite that they were developed in the early days of *Kp* QSAR development, e.g., the Potts and Guy model developed in 1992. Table 5 summarizes, for the *Kp* QSAR developed in this study, as well as for ten previously established *Kp* QSARs reviewed by Lian et al. [13] and Fitzpatrick et al. [34], the data used in the development of *Kp* QSAR, the number of descriptors included in the QSAR, and the fitting ability and predictive capability of the model. The models reported in Lien and Gao (1995) [16], Barratt (1995) [17], Potts and Guy (1995) [18], Abraham et al. (1995) [19], and Abraham et al. (1999) [20] were built from a dataset smaller than the one adopted in the current study, and thus reported high *R*^2^ in their original development processes. The influence of uneven sample size to the determination of *R*^2^ became evident when these models were re-validated by Lian et al. using a consistent dataset of 124 compounds. This validating dataset likely overlapped to various extents with the original datasets by which these models were developed. Nonetheless, in the validation these models were found to be of poor predictive capability, with a *Q*^2^ value ranging from 0.36–0.56, dropping significantly from the range of 0.90 to 0.96, as summarized in Fitzpatrick et al. The model by Potts and Guy in 1992 [15] was developed using a database comparable to the one adopted in this study, but the model was reported initially with an *R*^2^ of only 0.67. In the re-validation by Lian et al., a similar level of predictive power was observed.

A larger dataset of experimental *Kp* (158 compounds) was considered in Patel et al. [21]. One-hundred compounds in this dataset overlapped with those in the dataset applied for *Kp* QSAR development in this study. In Patel et al., the predictive capability of QSAR was not investigated, i.e., all of the 158 compounds in the dataset were used in model training. Among the *Kp* QSARs developed in Patel et al., the first model consisted of four descriptors and was of a *R*^2^ of 0.76 (Table 5, Equation (4)). Six steroid compounds, including hydrocortisone hemipimelate, hydrocortisone hemisuccinate, hydrocortisone hexanoate, hydrocortisone octanoate, hydrocortisone propinate, and hydrocortisone, were determined to be outliers to this four-descriptor model and removed from the original dataset. The second model was subsequently built using the 152 compounds remaining in the dataset and shown with an *R*^2^ of 0.83 (Table 5, Equation (5)). A further examination revealed an additional nine outliers among the 152 compounds used in building the second model. These outliers were removed from the dataset, and a third model was developed. The *R*^2^ of the third and final model increased to 0.90 for the remaining 143 compounds, and no additional outliers were identified (Table 5, Equation (6)). While the *R*^2^ in the final model of Patel et al. appeared to be higher than the level observed for the model developed in our study, it would be difficult to determine the applicability of the final model from Patel et al. in terms of its predictive power toward unknown compounds―it was unclear as to how the removal of outliers in the development of QSARs in Patel et al. might have impacted on the interpretation of structural characteristics or molecular mechanisms involved in transepidermal transport of the solutes. As previously described, a proper validation of QSAR was required before the model could be considered for regulatory application [35,36], and as such, the models presented in Patel et al. might be limited from such application given the lack of sufficient validation. To meet the requirement for regulation application, in the current study the *Kp* values included in the dataset for the development of *Kp* QSAR were randomly divided into a training dataset and a validation dataset at a ratio of 4 to 1. The *Q*^2^ identified for the current QSAR was 0.84, by far the highest value among the models compared in Table 5. In addition, there were no outliers identified from the 106 compounds in the dataset for developing the current *Kp* QSAR. These findings attest to the fitting and predictive capability of the proposed model.

### 2.4. Molecular Interactions Underlying Transepidermal Permeation of Small Molecules

**Figure 3:**
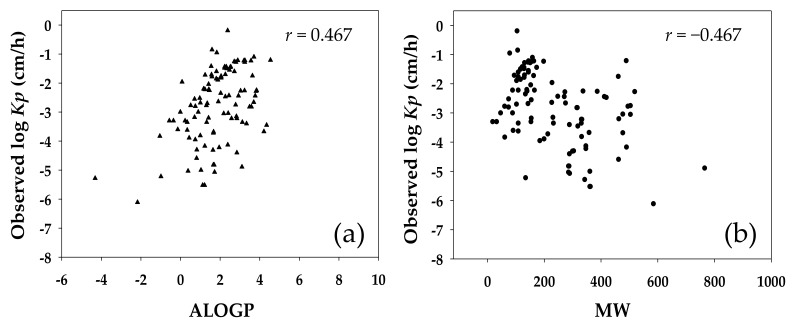
Distribution of experimentally observed logarithmic skin permeability (observed log *Kp*) against (**a**) Ghose-Crippen octanol-water partition coefficient (ALOGP) and (**b**) molecular weight (MW) for 106 compounds included in model training and validation datasets. The *r* value is Pearson product-moment correlation coefficient and describes linear dependence of observed log *Kp* to targeted descriptor.

The log *Kp* QSAR developed in this study identified four descriptors as significant molecular characteristics that affected the epidermal transport of small molecules. These descriptors exerted their influences via: (1) molecular cyclicity for single rings in the molecule reflecting local geometrical environments in complex cyclic systems (the descriptor D/Dr10); (2) sum of topological distances between all pairs of oxygen and chlorine atoms (T(O..Cl)); (3) partitioning of molecules between the lipophilic vs. hydrophilic phases of transport medium (ALOGP); and (4) antineoplastic-like property at 80% similarity (Neoplastic-80) (Table 2). As discussed, in the early days, the *Kp* QSARs were frequently established assuming a linear correlation between the log *Kp* and the regressor variables presumably indicative of lipophilicity and molecular size of a compound. The *Kp* in the models reported in Potts and Guy (1992) [15], Lien and Gao (1995) [16], and Patel et al. (2002) [21] (Table 1) was statistically related to the *K_OW_* and MW; while in the model developed by Barratt (1995) [17] the *Kp* was correlated to the *K_OW_* and MV. These QSARs shared a feature―the physicochemical descriptors *K_OW_* and MW/MV were combined and included in the model to suggest a mechanistic relevance of lipophilicity and molecular size to the transdermal transport of the solutes. In the current study, the molecular descriptor Ghose-Crippen octanol-water partition coefficient (ALOGP) was incorporated in the model, supporting the inference of lipophilicity being a key factor in the permeation of small molecules across the skin membrane. Figure 3 shows the distribution of the log value of experimentally determined *Kp* for the investigated compounds against their ALOGP and MW. The experimental *Kp* was moderately correlated to both the ALOGP and MW, conforming to the expected involvement of lipophilicity and molecular size in the dermal transport of small molecules. However, in the *Kp* QSAR established in this study the MW was selected only indirectly in the final array of molecular descriptors.

The exclusion of MW as a principal descriptor in the current *Kp* QSAR did not rule out the molecular size as a factor that contributed to the percutaneous permeation of small molecules. In the current QSAR, the molecular descriptor Ghose-Viswanadhan-Wendoloski antineoplastic-like index at 80% (Neoplastic-80) was selected to describe the transport of molecules through the stratum corneum. To the best of our knowledge, this is the first time that a composite index has been included as a descriptor in a *Kp* QSAR. The drug property-related indices have been applied widely in the evaluation of compounds of toxicological or pharmacological potency. For example, pharmaceuticals have frequently been evaluated for their therapeutic index, a ratio of the dose required to produce a toxic effect to the dose needed to elicit the desired therapeutic response [37] when a dose-response relationship was described. The comparison of the therapeutic effect versus the toxicological effect in a descriptive relationship of quantitative continuity is made on the recognition that many drugs share the same mechanisms of intercellular and intracellular transport as that of toxicants, or, in a broader sense, those of xenobiotics. In this study, the selection of a drug-related index in the *Kp* QSAR was perhaps an indication that many of the small-molecular-size compounds included in the dataset in this study exhibited behaviors of transport similar to those of antineoplastic compounds when moving across the stratum corneum. Further examination of the criteria adopted in determining the 80% similarity of a compound to antineoplastics [24] revealed a sub-domain of antineoplastic properties, including specific ranges of log *K_OW_*, molar refractivity (AMR), MW, and number of atoms in the molecule (nAT). Chemicals of log *K_OW_*, AMR, MW, and nAT values sitting in this sub-domain would be considered as sharing a similarity of 80% to an antineoplastic and subsequently assigned a value of 1 in the dichotomous index, whereas those of the aforementioned properties outside the sub-domain would be assigned a value of 0. Table 6 shows the ranges of log *K_OW_*, AMR, MW, and nAT defining the Neoplastic-80 as specified in Dragon^®^ and those corresponding to the 106 compounds included in the dataset in this study. For the compounds applied in the current study, the range of log *K_OW_*, AMR, MW, and nAT covered those that were specified in the antineoplastic sub-domain, resulting in the inclusion of Neoplastic-80 as a significant descriptor in the final model. Evidently, the MW was considered for its impact on the transepidermal transport of the solutes in the current QSAR, however, only when it was integrated as a part of a comprehensive scheme of influence and weighted with the other properties.

A primary goal in this study was, through the process of *Kp* QSAR development and a comparison with representative *Kp* QSARs in literature developed using a comparable database, to identify the evolution in molecular representation that best described the transdermal permeation behavior of molecules. This approach allowed us the opportunity to gain insights on the molecular characteristics affecting the transport of molecules in the epidermal skin. The identification of the descriptor Neoplastic-80 and its sub-domain consisting of antineoplastic properties as being relevant to the skin permeability of compounds in this study attests to this purpose. A more recent database reported in Baba et al. [31] collected experimentally derived permeability coefficients of 211 compounds consisting exclusively of permeability coefficients generated using an in vitro diffusion system of excised human skin. The aqueous donor solution in the diffusion system contained no organic solvents or permeation enhancers. This database was considered more consistent, as various criteria (e.g., requirements on in vitro study, use of human skin, use of aqueous vehicle, etc.) were applied in the process of *Kp* review, and recognized as more applicable to somewhat structurally complex compounds [32]. However, this database was not adopted in this study, as a primary goal in the current study was to compare between the *Kp* model developed in this study with representative QSAR models developed using similar and comparable databases. The *Kp* values generated for chemicals present in the solvent vehicle [38] or in a state of ionization [33] were not considered in this study neither, as the percutaneous absorption behavior of the molecules in these states could be a mixed result of influences from molecular properties as well as from a compromised integrity in the dermal barrier functions.

The *Kp* QSAR developed in this study is also one of potential for practical application. In the current *Kp* QSAR, the descriptors ALOGP and Neoplastic-80 are molecular property descriptors while D/Dr10 and T(O..Cl) are topological ones. For Neoplastic-80, a positive identification is made when the values of log *K_OW_*, AMR, MW, and nAT in the molecule of the compound fall in the ranges specified in the sub-domain (Table 6). The values of the properties in the sub-domain are available from online databases or reports in literature, and if necessary there are also algorithms amenable to the users for their calculation. For the topological descriptors, their values may also be derived following straightforward calculations, if an estimation using computer software or programs is unavailable. For example, the value of T(O..Cl) for the compound 2,4-dichlorophenol (CAS 120-83-2) is calculated as the sum of the topological distance from the chlorine in the *ortho* position of phenol to the hydroxyl group (3) and the distance from the chlorine in the *para* position to the hydroxyl group (5), yielding a final value of 8. The *Kp* QSAR developed in this study should serve as one of application potential with new perspectives on the molecular behaviors of compounds moving across the skin membrane.

In recent years, machine learning algorithms, such as artificial neural networks (ANN) and support vector machines, have been applied in developing predictive models. However, these methods may not be readily applied to building QSAR models from limited data. For example, the ANN has been frequently applied in developing nonlinear models for predicting skin permeability of chemicals [38,39,40]. When developing a three-layer ANN, assuming an input layer of simply five inputs (descriptors), a hidden layer of 10 nodes, and an output layer of one output (target property), the total number of parameters (weights and bias) in the ANN model will be 71 (5 × 10 + 10 + 10 × 1 + 1). As it is commonly required in the development of a predictive model that the ratio of the number of parameters in the model to the number of samples in the supporting dataset to be less than one-fifth, the number of parameters as estimated in this case inevitably results in overfitting the samples in our *Kp* dataset. Baba et al. [31] also commented that the ANNs were likely to overfit the given data and be trapped in local minima. In addition, their network structures could not be fully determined. In comparison, an MLR model of five descriptors would require the generation of only six parameters, which would make the MLR model much more amenable to the users. Considering the size of the dataset available in this study, the MLR was adopted as the mechanism in the QSAR development.

## 3. Materials and Methods

### 3.1. Skin Permeability Data

The *Kp* values of 106 structurally diverse compounds of anthropogenic or natural origins, as initially reported in Flynn [27] and Wilschut et al. [22], were selected and applied in the development of *Kp* QSAR in this study. These *Kp* values were determined using human cadaver skin and derived for compounds present in an aqueous vehicle. The *Kp* values in these databases were predominantly derived from in vitro studies. The values for benzene, styrene, and toluene in the Flynn database were reported to be derived from in vivo measurements [41]. The precise procedures or experimental details in determining these *Kp* were not reported in the original databases. Consequently, inter-laboratory uncertainty and methodological variation in the *Kp* were expected. Despite the ambiguity inherent in these *Kp* arising from experimental variation, the use of *Kp* only for human skin reduced the complexity involved in animal-to-human interpretation of the experimental *Kp*. In addition, the *Kp* values in these two databases have been the primary source of data used in support of the development for human *Kp* QSARs, e.g., the models developed by Potts and Guy (1992) [15], Potts and Guy (1995) [18], Patel et al. (2002) [21], Lien and Gao (1995) [16], Barratt (1995) [17], Abraham et al. (1995) [19], and Abraham et al. (1999) [20]. Their adoption in the current study made available the opportunity to observe the change in the selection of molecular descriptors for the model developed in this study from the strategies of descriptor selection applied in the aforementioned *Kp* QSARs. The Chemical Abstract Service (CAS) number, chemical name, log *Kp*, MW, log *K_OW_*, and stage of application in the model development for the 106 compounds included in this study are summarized and provided in Table S1.

### 3.2. Partitioning of Skin Permeability Data for Model Training and Validation

**Figure 4:**
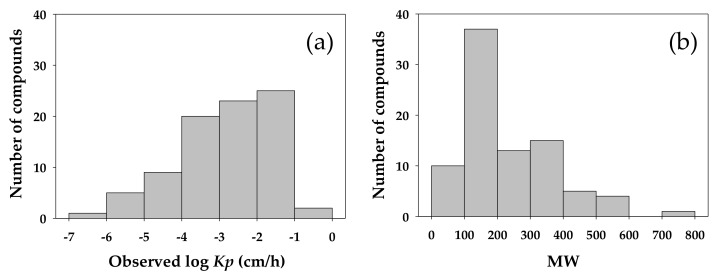
Abundance and distribution of compounds in training dataset for model development as arranged and displayed by (**a**) logarithmic value of experimentally determined skin permeation coefficient (observed log *Kp*) and (**b**) molecular weight (MW) of compound. A total of 85 compounds were included in training dataset.

**Figure 5:**
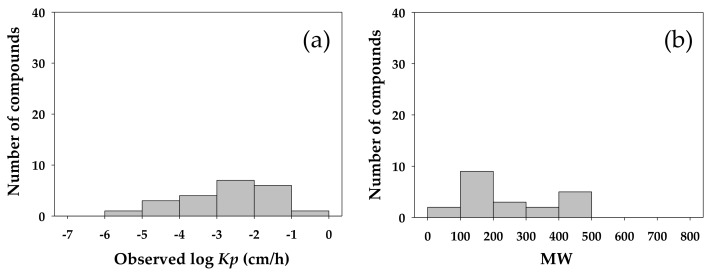
Abundance and distribution of compounds in validation dataset for model development as arranged and displayed by (**a**) logarithmic value of experimentally determined skin permeation coefficient (observed log *Kp*) and (**b**) molecular weight (MW) of compound. A total of 21 compounds were included in validation dataset.

**Figure 6:**
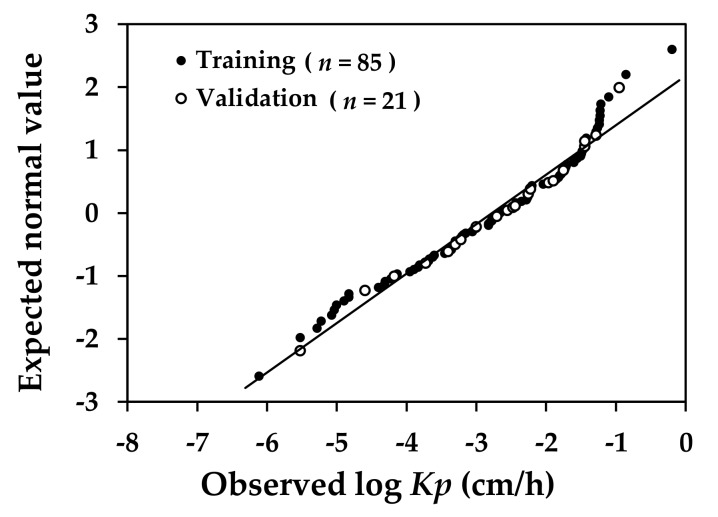
Normal probability plot for logarithmic values of experimentally determined skin permeation coefficient (observed log *Kp*) of compounds included in training and validation datasets.

The logarithmic values of experimental *Kp* and the values of MW for the 106 candidate compounds selected in this study ranged from –6.11 to –0.19 and from 18.0 to 764.9, respectively. These compounds were randomly partitioned into a training set of 85 compounds and a validation set of 21. The number of compounds in the validation set was about one-fifth of its counterpart in the training set, a ratio recommended for the validation of an empirical model [25,42]. The log *Kp* and MW for the training compounds ranged from –6.11 to –0.19 and from 18.0 to 764.9, respectively, while those for the validation compounds ranged from –5.52 to –0.96 and from 46.1 to 489.6. To evaluate if the compounds included in the validation set were representative of those in the training set, the values of log *Kp* and MW of the compounds were distributed in histogram for those included in the training set (Figure 4) and in the validation set (Figure 5). The comparison in log *Kp* and MW between the training and validating compounds served to indicate the relevance between these two groups of data in describing the behavior of compounds permeating across the skin membrane. The MW was included in this comparison as this property has been long recognized as a readily measurable physicochemical property well correlated to the transdermal penetration behavior of solutes and examined in the *Kp* QSAR development [22,31]. In addition, the MW commonly served to suggest biological activity of molecules in the development of QSARs targeting toxicological endpoints. For example, Lei et al. [43] examined and compared the chemical distribution of molecules in the training and validation datasets as defined by the MW and the Wildman and Crippen’s octanol-water partition coefficient in their prediction of the acute toxicity by route of oral exposure. As the comparison between Figure 4a and Figure 5a revealed, the distribution of log *Kp* in both the training and validation set in this study was comparable, suggesting that the validation data was a representative subset of the training dataset. In the literature [22,23], the log *Kp* of chemical was identified to be negatively correlated to the MW. A similar trend was identified in this study. The experimental *Kp* values of the compounds in model training and validation datasets were also examined for normality in their distribution. As shown in Figure 6, these data conformed to the assumption of normality for being applied to developing the *Kp* QSAR via the MLR technique.

### 3.3. Molecular Structure Construction and Optimization

As the first step of model development, molecular structure files were constructed for each individual compound included in the training and validation datasets. For the 106 compounds included in this study, the molecular structures were extracted mainly from the US National Library of Medicine TOXNET ChemIDplus Database (TOXNET) [44]. In rare cases where the files were not available from the TOXNET, they were extracted from the National Institute of Standards and Technology’s Chemistry WebBook [45] and SciFinder^®^ [46]. These molecular structure files were graphically transformed and optimized in the HyperChem^®^ Molecular Modeling System [47]. The molecular mechanics calculations involved in the optimization were performed first using MM+ force field to optimize the molecular geometries with lower optimization accuracy, and then the semi-empirical calculations were carried out using the routines AM1 to complete the full geometry optimization with higher accuracy.

### 3.4. Molecular Descriptor Calculation

In the next step, the Dragon^®^ software [24] in version 5.5 was used to calculate the molecular descriptors for all the compounds according to their optimized molecular structure. A newer release of Dragon^®^ was available and had been applied in the development of QSAR. For example, Chavan et al. [48] employed Dragon^®^ 6.0 in their investigation of the possibility of establishing a global QSAR model for acute toxicity based on a database of 436 chemicals. In this study, however, version 5.5 of the software was employed, as it allowed us to follow the definition and algorithm underlying the calculation of specific molecular descriptors in order to characterize the molecular properties with these descriptors, which was essential to interpreting the transdermal movement behavior of molecules in the epidermal skin. The definition and relevant algorithmic details for all molecular descriptors in Dragon^®^ 5.5 may be found, as indicated in its user manual, in the *Handbook of Molecular Descriptors* [49] and the *Molecular Descriptors for Chemoinformatics* [50]. The Dragon^®^ software in version 5.5 could calculate up to 3,224 descriptors for every molecule. However, some molecular descriptors gave the same numerical values for all explored compounds, and in the MLR model they were indistinguishable in terms of a correlation with the dependent variable for the explored dataset. As a result these descriptors were excluded from the MLR model construction. With their removal a total of 1530 molecular descriptors remained as the candidates of the regressor variables for the MLR model of log *Kp*.

### 3.5. Molecular Descriptor Selection and Multiple Linear Regression

When an MLR model was developed from a large number of regressor variables, the correlations between regressor variables were assessed to prevent the inclusion of redundant regressor variables in the model and a consequent reduction in the explanatory power of individual regressor variable. By the same principle, in the current study the optimal *Kp* QSAR would be the one that ultimately predicted log *Kp* using the least number of molecular descriptors and with the highest accuracy. The process of selecting a subset of regressor variables, in this study the molecular descriptors, for creating a model with fewer regressors was often referred to as the feature selection in the literature [42]. A typical criterion applied in this selection process is the minimization of a specific measure of predictive error for an investigated model. A variety of algorithms have been proposed to search for a specific subset of regressors that optimally model the measured response under the constraints of required or excluded features, size of subset, etc. The stepwise regression algorithm was adopted in this study to provide a systematic method for adding and removing regressor variables based on their statistical significance in a regression model [42]. This method began with one initially given model and compared the explanatory power of incrementally larger and smaller regressor variables when they were fit into this model. At each step, the *p* value of the *F* statistic was computed to test the model with and without a potential regressor. If a regressor to be tested was not yet included in the model, the null hypothesis would be that this regressor had a zero coefficient when it was added to the model. If there was sufficient evidence to reject the null hypothesis, the regressor variable was added. Conversely, if a regressor was currently present in the model, then the null hypothesis was that the regressor had a zero coefficient. If there was insufficient evidence to reject the null hypothesis, the regressor variable was removed from the model. However, depending on the regressor variables included in the initial model and the order in which the variables were moved in and out, this step regression might build up different models from the same set of potential regressor variables. In this sense, the model generated by the stepwise regression was a locally optimal model instead of a globally optimal one [42]. To overcome this drawback, in the current study, the random search technique was combined into the stepwise regression algorithm in finding the regressor variables. This modified algorithm automatically chose the regressor variables of higher correlation coefficient as the possible candidates for input into the initial model in the conventional stepwise regression algorithm [51,52]. The calculated values of molecular descriptors incorporated in the established model and the log *Kp* predicted by the model for the 106 compounds included in this study are summarized in Table S2.

## 4. Conclusions

The rate of chemicals permeating through the stratum corneum, the *Kp*, is widely used to quantitatively describe the potential of dermal absorption for manmade and naturally derived compounds. The regulatory application of the *Kp* in standard development, however, is frequently hindered by the lack of *Kp* values that are experimentally determined in accordance with standardized and consistent protocols. This study developed a four-descriptor *Kp* QSAR based on 106 compounds of *Kp* determined using human skin. Similar to the models reported in the literature, the *Kp* QSAR developed in the current study considered log *K_OW_* a direct influence on the transdermal permeation of small molecules. This *Kp* model identified, for the first time in the QSAR literature, a sub-domain of molecular properties initially defined to describe the antineoplastic resemblance of a compound as a significant factor in the permeation of a solute through the skin membrane. This finding suggested that the influence of molecular size on the skin permeation of chemicals should be interpreted with other physicochemical properties, rather than being represented by molecular weight alone. The *Kp* QSAR reported in this study may serve as a source of *Kp* in support of dermal hazard characterization when experimentally determined *Kp* values are not readily available.

## Figures and Tables

**Table 1:** Evolution of quantitative structure-activity relationship developed for predicting skin permeability ^a^.

Model (Year)	QSAR and molecular descriptors ^b^
Potts and Guy (1992) [15]	$\log\;Kp\;(\mathrm{cm}/s)\;=\;0.71{\log\;K}_{OW}\;-\;0.0061\;\mathrm{MW}\;-\;6.3$
Lien and Gao (1995) [16]	$\log\;Kp\;(\mathrm{cm}/s)\;=\;0.84{\log\;K}_{OW}\;-\;0.07{(\log\;K}_{OW}{)}^{2}\;-\;0.27H_{b}\;-\;\;1.84\;\log\;\mathrm{MW}\;+\;0.8337$
Barratt (1995) [17]	$\log\;Kp\;(\mathrm{cm}/s)\;=\;0.82{\log\;K}_{OW}\;-\;0.0093\;\mathrm{MV}\;-\;0.039\;\mathrm{MPt}\;-\;5.9163$
Potts and Guy (1995) [18]	$\log\;Kp\;(\mathrm{cm}/s)\;=\;0.0256\;\mathrm{MV}\;-\;1.72\sum^{\text{​}}\alpha_{2}^{H}-\;3.93\;\sum^{\text{​}}\beta_{2}^{H}-\;4.85$
Abraham et al. (1995) [19]	$\log\;Kp\;(\mathrm{cm}/s)\;=\;-\;0.59\pi_{2}^{H}\;-\;0.63\sum^{\text{​}}\alpha_{2}^{H}-\;3.48\;\sum^{\text{​}}\beta_{2}^{H}+\;1.79V_{x}\;-\;5.05$
Abraham et al. (1999) [20]	$\log\;Kp\;(\mathrm{cm}/s)\;=\;0.44R_{2}\;-\;0.49\pi_{2}^{H}\;-\;1.48\sum^{\text{​}}\alpha_{2}^{H}-\;3.44\;\sum^{\text{​}}\beta_{2}^{H}\;+\;1.94V_{x}\;-\;5.13$
Patel et al. (2002) [21]	$\log\;Kp\;=\;0.681\;\log\;K_{OW}\;-\;0.00653\;\mathrm{MW}\;-\;0.284\;\mathrm{ABSQon}\;-\;\;0.268\;\mathrm{SsssCH}\;-\;2.47$
Mitragotri (2002) [14]	$P\;=\;5.6\times \;10^{-6}{\;K}_{o/w}^{0.7}\;\exp(-0.46r^{2})$

^a^ The Potts and Guy (1992), Lien and Gao (1995), Barratt (1995), Potts and Guy (1995), Abraham et al. (1995), and Abraham et al. (1999) were adopted as summarized in Lian et al. [13]. ^b^ QSAR = quantitative structure-activity relationship; *Kp* (*P*) = skin permeability; *K_OW_* = octanol-water partition coefficient; MW = molecular weight; *H_b_* = number of hydrogen bonds; MV = molecular volume; MPt = melting point; $\sum^{\text{​}}\alpha_{2}^{H}$ = solute hydrogen bond acidity; $\sum^{\text{​}}\beta_{2}^{H}$ = solute hydrogen bond basicity; $\pi_{2}^{H}$ = solute dipolarity/polarisability; *V_x_* = McGowan characteristic molecular volume; *R*_2_ = excess molar refraction; ABSQon = the sum of absolute charges on oxygen and nitrogen atoms; SsssCH = the sum of E-state indices for all methyl groups; *K_o/w_* = octanol-water partition coefficient; *r* = solute molecular radius in Angstroms (Å).

**Table 2:** Molecular descriptors in quantitative structure-activity relationship developed for prediction of skin permeability.

Molecular Descriptor (Dragon^®^ Name)	Type	Definition
D/Dr10	Topological descriptor	Distance/detour ring index of order 10
T(O..Cl)	Topological descriptor	Sum of topological distances between O..Cl
ALOGP	Molecular property	Ghose-Crippen octanol-water partition coefficient (log *P*)
Neoplastic-80	Molecular property	Ghose-Viswanadhan-Wendoloski antineoplastic-like index at 80%

**Table 3:** Analysis of variance testing fitting ability of quantitative structure-activity relationship to training dataset ^a^.

	*df*	*SS*	*MS*	*F*	*p* Value
Regression	4	117.399	29.350	121.287	0.000
Residue	80	19.359	0.242		
Sum	84	136.758			

^a^ *df* = degrees of freedom; *SS* = sum of squares; *MS* = mean square; *F* = *F* statistic.

**Table 4:** Fitting ability and predictive capability of quantitative structure-activity relationship for estimating skin permeability ^a^.

Dataset	MSE	*R*^2^/*Q*^2^	AME	AAE
Training	0.228	0.858	1.582	0.344
Validation	0.206	0.839	1.081	0.345

^a^ MSE = mean square error; *R*^2^/*Q*^2^ = coefficients of determination; AME = absolute maximum error; AAE = average absolute error.

**Table 5:** Performance of skin permeability model from this study and of those reviewed in literature ^a,b^.

*Kp* QSAR Model	Model Development				Model Validation		Remarks
	*n_d_*	*n_s_*	*R*^2^	Source(s) of Experimental *Kp* ^c^	*Q*^2^	MAE	
Potts and Guy (1992) [15]	2	93	0.67	Flynn	0.68	0.091	^d,e^
Lien and Gao (1995) [16]	4	22	0.96	Flynn	0.56	0.402	^d,e^
Barratt (1995) [17]	3	60	0.90	Flynn	0.46	0.632	^d,e^
Potts and Guy (1995) [18]	3	37	0.94	Flynn	0.36	0.274	^d,e^
Abraham et al. (1995) [19]	4	46	0.96	Flynn	0.54	0.140	^d,e^
Abraham et al. (1999) [20]	5	53	0.96	Flynn	0.54	0.120	^d,e^
Patel et al. (2002), Equation (4) [21]	4	158	0.76	Flynn, Wilschut et al.	n.a.	n.a.
Patel et al. (2002), Equation (5) [21]	4	152	0.83	Flynn, Wilschut et al.	n.a.	n.a	^f^
Patel et al. (2002), Equation (6) [21]	4	143	0.90	Flynn, Wilschut et al.	n.a.	n.a	^g^
The current study	4	85	0.86	Flynn, Wilschut et al.	0.84	0.206	

^a^ *Kp* = skin permeability; QSAR = quantitative structure-activity relationship; *n_d_* = number of descriptors; *n_s_* = number of compounds; *R*^2^/*Q*^2^ = coefficients of determination; MAE = mean absolute error. ^b^ Previously established *Kp* QSARs were reviewed and evaluated in Lian et al. [13] and Fitzpatrick et al. [34]. ^c^ Experimental *Kp* reported in Flynn [27] and Wilschut et al. [22] were those determined using human epidermal skin. ^d^ Information of model development was extracted from Fitzpatrick et al. ^e^ A dataset consisting of 205 *Kp* values originating from 124 chemical compounds was applied consistently to these six models in the re-determination of *R*^2^ in Lian et al. ^f^ Six steroid compounds including hydrocortisone hemipimelate (CAS No. 107085-84-7), hydrocortisone hemisuccinate (CAS No. 2203-97-6), hydrocortisone hexanoate (CAS No. 3593-96-2), hydrocortisone octanoate (CAS No. 6678-14-4), hydrocortisone propinate (CAS No. 6677-98-1), and hydrocortisone (CAS No. 50-23-7) were determined to be outliers to Equation (4) in Patel et al. and removed from the original 158 compounds when developing Equation (5). ^g^ Nine compounds including atropine (CAS No. 51-55-8), benzaldehyde (CAS No. 100-52-7), diclofenac (CAS No. 15307-86-5), digitoxin (CAS No. 71-63-6), estriol (CAS No. 50-27-1), etorphine (CAS No. 14521-96-1), indomethacin (CAS No. 53-86-1), naproxen (CAS No. 22204-53-1), and nicotine (CAS No. 54-11-5) were determined to be outliers to Equation (5) in Patel et al. and further deleted from the remaining 152 compounds when developing Equation (6).

**Table 6:** Ranges of molecular descriptors for sub-domain of antineoplastic properties and those for model-developing compounds ^a^.

Dataset	log *K_OW_*	AMR	MW	nAT
Sub-domain defined in Dragon^®^ for Neoplastic-80 [24]	−1.5 to 4.7	43 to 128	180 to 475	21 to 63
Range observed in compounds included in model development	−3.1 to 5.5	3 to 192	18 to 765	3 to 118

^a^ Log *K_OW_* = logarithmic octanol-water partition coefficient; AMR = molar refractivity; MW = molecular weight; nAT = number of atoms in the molecule; Neoplastic-80 = Ghose-Viswanadhan-Wendoloski antineoplastic-like index at 80%.

## Supplementary Materials

The following are available online. Table S1: List of compounds applied in development of quantitative structure-activity relationship for skin permeability prediction; Table S2: List of values estimated for molecular descriptors and skin permeability using quantitative structure-activity relationship developed in this study for compounds applied in model training and validation.

## References

[1] Sartorelli P., Ahlers H.W., Alanko K., Chen C.-P., Cherrie J.W., Drexler H., Kezic S., Johanson G., Filon F.L., Maina G. (2007). How to improve skin notation. Position paper from a workshop. Regul. Toxicol. Pharmacol..

[2] Dotson G.S., Chen C.-P., Gadagbui B., Maier A., Ahlers H.W., Lentz T.J. (2011). The evolution of skin notations for occupational risk assessment: A new NIOSH strategy. Regul. Toxicol. Pharmacol..

[3] American Conference of Governmental Industrial Hygienists (ACGIH) (2013). Documentation of the TLVs® and BEIs® with Other Worldwide Occupational Exposure Values.

[4] Chen C.-P., Ahlers H.W., Dotson G.S., Lin Y.-C., Chang W.-C., Maier A., Gadagbui B. (2011). Efficacy of predictive modeling as a scientific criterion in dermal hazard identification for assignment of skin notations. Regul. Toxicol. Pharmacol..

[5] Organisation for Economic Co-operation and Development (OECD) (2004). OECD Series on Testing and Assessment No. 28: Guidance Document for the Conduct of Skin Absorption Studies.

[6] Organisation for Economic Co-operation and Development (OECD) (2004). OECD Guideline for Testing of Chemicals 428: Skin Absorption—In Vitro Method.

[7] U.S. Environmental Protection Agency (USEPA) (2004). In Vitro Dermal Absorption Rate Testing of Certain Chemicals of Interest to the Occupational Safety and Health Administration. Final Rule. 69 Federal Register 22402.

[8] National Institute for Occupational Safety and Health (NIOSH) (2009). A Strategy for Improvement of Skin Notations.

[9] Walker J.D., Rodford R., Patlewicz G. (2003). Quantitative structure-activity relationship for predicting percutaneous absorption rates. Environ. Toxicol. Chem..

[10] European Centre for Ecotoxicology and Toxicology of Chemicals (ECETOC) (1998). Examination of a Proposed Skin Notation Strategy.

[11] Walker J.D., Whittaker C., McDougal J.N., Marzulli F.N., Maibach H.I. (1996). Role of the TSCA Interagency Testing Committee in meeting the U.S. government data needs: Designating chemicals for percutaneous absorption rate testing. Dermatotoxicology.

[12] Frasch H.F., Dotson G.S., Bunge A.L., Chen C.-P., Cherrie J.W., Kasting G.B., Kissel J.C., Sahmel J., Semple S., Wilkinson S. (2014). Analysis of finite dose dermal absorption data: Implications for dermal exposure assessment. J. Expo. Sci. Environ. Epidemiol..

[13] Lian G., Chen L., Han L. (2008). An evaluation of mathematical models for predicting skin permeability. J. Pharm. Sci..

[14] Mitragotri S. (2002). A theoretical analysis of permeation of small hydrophobic solutes across the skin based on Scaled Particle Theory. J. Pharm. Sci..

[15] Potts R.O., Guy R.H. (1992). Predicting skin permeability. Pharm. Res..

[16] Lien E.J., Gao H. (1995). QSAR analysis of skin permeability of various drugs in man as compared to in vivo and in vitro studies in rodents. Pharm. Res..

[17] Barratt M.D. (1995). Quantitative structure-activity relationships for skin permeability. Toxicol. In Vitro.

[18] Potts R.O., Guy R.H. (1995). A predictive algorithm for skin permeability: The effects of molecular size and hydrogen bond activity. Pharm. Res..

[19] Abraham M.H., Chadha H.S., Mitchell R.C. (1995). The factors that influence skin penetration of solutes. J. Pharm. Pharmacol..

[20] Abraham M.H., Chadha H.S., Martins F., Mitchell R.C., Bradbury M.W., Gratton J.A. (1999). Hydrogen bonding part 46: A review of the correlation and prediction of transport properties by an LFER method: Physicochemical properties, brain penetration and skin permeability. Pestic. Sci..

[21] Patel H., ten Berge W., Cronin M.T.D. (2002). Quantitative structure-activity relationships (QSARs) for the prediction of skin permeation of exogenous chemicals. Chemosphere.

[22] Wilschut A., ten Berge W.F., Robinson P.J., McKone T.E. (1995). Estimating skin permeation. The validation of five mathematical skin permeation models. Chemosphere.

[23] Mitragotri S., Anissimov Y.G., Bunge A.L., Frasch H.F., Guy R.H., Hadgraft J., Kasting G.B., Lane M.E., Roberts M.S. (2011). Mathematical models of skin permeability: An overview. Int. J. Pharm..

[24] Talete srl (2007). DRAGON for Windows and Linux 2007: DRAGON User Manual Version 5.5.

[25] Eriksson L., Johansson E., Kettaneh-Wold N., Trygg J., Wikström C., Wold S. (2006). Multi- and Megavariate Data Analysis Part I Basic Principles and Applications.

[26] Van de Sandt J.J.M., van Burgsteden J.A., Cage S., Carmichael P.L., Dick I., Kenyon S., Korinth G., Larese F., Limasset J.C., Maas W.J.M. (2004). In vitro predictions of skin absorption of caffeine, testosterone, and benzoic acid: A multi-centre comparison study. Regul. Toxicol. Pharmacol..

[27] Flynn G.L., Gerrity T.R., Henry C.J. (1990). Physicochemical determinants of skin absorption. Principles of Route-to-Route Extrapolation for Risk Assessment.

[28] Bronaugh R.L., Congdon E.R., Scheuplein R.J. (1981). The effect of cosmetic vehicles on the penetration of *N*-nitrosodiethanolamine through excised human skin. J. Investig. Dermatol..

[29] Franz T.J., Lehman P.A., Franz S.F., North-Root H., Demetrulias J.L., Kelling C.K., Moloney S.J., Gettings S.D. (1993). Percutaneous penetration of *N*-nitrosodiethanolamine through human skin (in vitro): Comparison of finite and infinite dose applications from cosmetic vehicles. Fundam. Appl. Toxicol..

[30] Brain K.R., Walters K.A., James V.J., Dressler W.E., Howes D., Kelling C.K., Moloney S.J., Gettings S.D. (1995). Percutaneous penetration of dimethylnitrosamine through human skin in vitro: Application from cosmetic vehicles. Food Chem. Toxicol..

[31] Baba H., Takahara J., Mamitsuka H. (2015). In silico predictions of human skin permeability using nonlinear quantitative structure–property relationship models. Pharm. Res..

[32] Lindh M., Karlén A., Norinder U. (2017). Predicting the rate of skin penetration using an aggregated conformal prediction framework. Mol. Pharm..

[33] Zhang K., Abraham M.H., Liu X. (2017). An equation for the prediction of human skin permeability of neutral molecules, ions and ionic species. Int. J. Pharm..

[34] Fitzpatrick D., Corish J., Hayes B. (2004). Modelling skin permeability in risk assessment―The future. Chemosphere.

[35] Quintero F.A., Patel S.J., Muñoz F., Mannan M.S. (2012). Review of existing QSAR/QSPR models developed for properties used in hazardous chemicals classification system. Ind. Eng. Chem. Res..

[36] European Chemicals Agency (ECHA) (2008). Chapter R6: QSARs and grouping of chemicals. Guidance on Information Requirements and Chemical Safety Assessment.

[37] Eaton D.L., Gilbert S.G., Klaassen C.D. (2013). Principles of toxicology. Casarett and Doull’s Toxicology―The Basic Science of Poisons.

[38] Atobe T., Mori M., Yamashita F., Hashida M., Kouzuki H. (2015). Artificial neural network analysis for predicting human percutaneous absorption taking account of vehicle properties. J. Toxicol. Sci..

[39] Katritzky A.R., Dobchev D.A., Fara D.C., Hür E., Tämm K., Kurunczi L., Karelson M., Varnek A., Solov’ev V.P. (2006). Skin permeation rate as a function of chemical structure. J. Med. Chem..

[40] Chen L.J., Lian G.P., Han L.J. (2007). Prediction of human skin permeability using artificial neural network (ANN) modeling. Acta Pharmacol. Sin..

[41] Vecchia B., Bunge A., Guy R.H., Hadgraft J. (2003). Skin absorption databases and predictive equations. Transdermal Drug Delivery.

[42] The MathWorks Inc. (2009). MATLAB Statistical Toolbox v7.7—User Guide.

[43] Lei T., Li Y., Song Y., Li D., Sun H., Hou T. (2016). ADMET evaluation in drug discovery: 15. Accurate prediction of rat oral acute toxicity using relevance vector machine and consensus modeling. J. Cheminform..

[44] U.S. National Library of Medicine: Toxicology Data Network (TOXNET) ChemIDplus Database. https://toxnet.nlm.nih.gov/.

[45] National Institute of Standards and Technology: Chemistry WebBook. http://webbook.nist.gov/chemistry/.

[46] American Chemical Society: SciFinder®—A CAS Solution. https://scifinder.cas.org/.

[47] Hypercube Inc. (2008). HyperChem® Release 8.0 for Windows®: Reference Manual.

[48] Chavan S., Nicholls I.A., Karlsson B.C.G., Rosengren A.M., Ballabio D., Consonni V., Todeschini R. (2014). Towards global QSAR model building for acute toxicity: Munro database case study. Int. J. Mol. Sci..

[49] Todeschini R., Consonni V. (2008). Handbook of Molecular Descriptors.

[50] Todeschini R., Consonni V., Mannhold R., Kubinyi H., Folkers G. (2009). Molecular Descriptors for Chemoinformatics.

[51] Tsai F.-Y., Chen C.-C., Liaw H.-J. (2012). A model for predicting the auto-ignition temperature using quantitative structure property relationship approach. Procedia Eng..

[52] Chen C.-P., Chen C.-C., Chen H.-F. (2014). Predicting flash point of organosilicon compounds using quantitative structure activity relationship approach. J. Chem..

