# Supplementary material for: Evaluating Molecular Properties Involved in Transport of Small Molecules in Stratum Corneum: A Quantitative Structure-Activity Relationship for Skin Permeability
Source: Molecules. 2018 Apr 15;23(4):911. doi: 10.3390/molecules23040911 (PMC6017021; doi:10.3390/molecules23040911)
Supplement: Supplementary file 1 [file molecules-23-00911-s001.pdf]

Supplementary Materials

Evaluating Molecular Properties Involved in Transport of Small Molecules in Stratum Corneum: A Quantitative Structure-Activity Relationship for Skin Permeability

Chen-Peng Chen [1], Chan-Cheng Chen [2,*], Chia-Wen Huang [1] and Yen-Ching Chang [1]

[1] Department of Occupational Safety and Health, College of Public Health, China Medical University, No. 91 Hsueh-Shih Road, Taichung 40402, Taiwan; chencp@mail.cmu.edu.tw (C.-P.C.); u100014014@cmu.edu.tw (C.-W.H.); yen071013@gmail.com (Y.-C.C.)

[2] Department of Safety, Health and Environmental Engineering, National Kaohsiung University of Science and Technology, No.1, University Road, Yanchao District, Kaohsiung City 824, Taiwan; chch_chen@nkfust.edu.tw

* Correspondence: chch_chen@nkfust.edu.tw; Tel.: +886-7-6011000, ext. 32311

Table S1. List of compounds applied in development of quantitative structure-activity relationship for skin permeability prediction.

Table S2. List of values estimated for molecular descriptors and skin permeability using quantitative structure-activity relationship developed in this study for compounds applied in model training and validation.

Table S1. List of compounds applied in development of quantitative structure-activity relationship for skin permeability prediction. Relevant information includes the CAS number, chemical name, logarithmic value of experimentally observed skin permeability (observed log *Kp*), molecular weight (MW), logarithmic value of octanol-water partition coefficient (log *Kow*), and stage of application in the model development for the 106 compounds included in the training and validation datasets.

CAS No.	Chemical Name	Observed log *Kp* (cm/h)	MW	log *Kow*	Modeling Stage
[71-55-6]	1,1,1-Trichloroethane	−2.35	133.40	2.49	Training
[88-06-2]	2,4,6-Trichlorophenol	−1.23	197.50	3.69	Training
[120-83-2]	2,4-Dichlorophenol	−1.22	163.00	3.06	Training
[99-57-0]	2-Amino-4-nitrophenol	−3.18	154.10	1.26	Training
[95-57-8]	2-Chlorophenol	−1.48	128.60	2.15	Training
[95-48-7]	*o*-Cresol	−1.80	108.10	1.95	Training
[110-80-5]	2-Ethoxyethanol	−3.60	90.10	−0.32	Training
[5307-14-2]	2-Nitro-*p*-phenylenediamine	−3.30	153.10	0.53	Validation
[95-54-5]	*o*-Phenylenediamine	−3.35	108.10	0.15	Training
[60-12-8]	2-Phenylethanol	−1.42	122.20	1.36	Training
[95-65-8]	3,4-Xylenol	−1.44	122.10	2.30	Validation
[108-39-4]	*m*-Cresol	−1.82	108.10	1.96	Training
[554-84-7]	3-Nitrophenol	−2.25	139.10	2.00	Validation
[119-34-6]	4-Amino-2-nitrophenol	−2.55	154.10	0.96	Validation
[106-41-2]	4-Bromophenol	−1.44	173.00	2.59	Training
[5131-60-2]	4-Chloro-*m*-phenylenediamine	−2.68	142.60	0.85	Training
[106-48-9]	4-Chlorophenol	−1.44	128.60	2.39	Validation
[106-44-5]	*p*-Cresol	−1.76	108.10	1.94	Training
[123-07-9]	4-Ethylphenol	−1.46	122.20	2.58	Training
[100-02-7]	4-Nitrophenol	−2.25	139.10	1.91	Training
[106-50-3]	*p*-Phenylenediamine	−3.62	108.10	−0.30	Training
[52-39-1]	Aldosterone	−5.52	360.40	1.08	Training
[57-43-2]	Amylobarbital	−2.64	226.30	2.07	Training
[100-66-3]	Anisole	−1.60	108.10	2.11	Training
[51-55-8]	Atropine	−5.07	289.40	1.83	Training
[57-44-3]	Barbital	−3.95	184.20	0.65	Training
[100-52-7]	Benzaldehyde	−0.85	106.10	1.48	Training
[71-43-2]	Benzene	−0.95	78.10	2.13	Validation
[100-51-6]	Benzyl alcohol	−2.22	108.10	1.10	Training
[135-19-3]	*b*-Naphtol	−1.55	144.20	2.70	Training
[77-28-1]	Butobarbital	−3.72	212.40	1.73	Validation
[107-92-6]	Butyric acid	−3.00	88.11	0.79	Training
[59-50-7]	Chlorocresol	−1.26	142.60	3.10	Training
[88-04-0]	Chloroxylenol	−1.23	156.60	3.27	Training
[132-22-9]	Chlorpheniramine	−2.66	274.80	3.38	Training
[76-57-3]	Codeine	−4.31	299.40	1.19	Training

[152-58-9]	Cortexolone	−4.13	346.50	3.08	Training
[64-85-7]	Cortexone	−3.35	330.50	2.88	Training
[50-22-6]	Corticosterone	−4.22	346.50	1.94	Training
[53-06-5]	Cortisone	−5.00	360.50	1.47	Training
[15307-86-5]	Diclofenac	−3.45	318.00	4.51	Training
[90-89-1]	Diethylcarbamazine	−3.89	199.30	0.37	Training
[71-63-6]	Digitoxin	−4.89	764.90	1.85	Training
[509-60-4]	Dihydromorphine	−4.82	287.36	0.93	Training
[299-42-3]	Ephedrine	−2.22	165.20	1.13	Validation
[50-28-2]	Estradiol	−2.28	272.40	4.01	Training
[50-27-1]	Estriol	−4.40	288.40	2.45	Training
[53-16-7]	Estrone	−2.44	270.40	3.13	Training
[64-17-5]	Ethanol	−3.00	46.10	−0.31	Validation
[60-29-7]	Ethyl ether	−2.80	74.10	0.89	Training
[14521-96-1]	Etorphine	−2.44	411.50	2.79	Validation
[437-38-7]	Fentanyl	−2.25	336.50	4.05	Training
[356-12-7]	Fluocinonide	−2.77	494.60	3.19	Training
[111-14-8]	Heptanoic acid	−1.70	130.19	2.42	Training
[53535-33-4]	Heptanol	−1.49	116.20	2.31	Training
[142-62-1]	Hexanoic acid	−1.85	116.16	1.92	Training
[107085-84-7]	Hydrocortisone hemipimelate	−2.75	503.60	3.26	Training
[2203-97-6]	Hydrocortisone hemisuccinate	−3.20	462.50	1.89	Training
[3593-96-2]	Hydrocortisone hexanoate	−1.75	460.60	4.48	Validation
[114593-88-3]	Hydrocortisone hydroxyhexanoate	−3.04	476.60	2.79	Training
[114611-36-8]	Hydrocortisone methylpimelate	−2.27	518.60	3.70	Training
[114593-86-1]	Hydrocortisone methylsuccinate	−3.68	476.60	2.60	Training
[74253-50-2]	Hydrocortisone *N,N*-dimethylsuccinamate	−4.17	489.60	2.03	Validation
[6678-14-4]	Hydrocortisone octanoate	−1.21	488.70	5.49	Training
[114593-87-2]	Hydrocortisone pimelamate	−3.05	503.60	2.31	Training
[6677-98-1]	Hydrocortisone propionate	−2.47	418.50	2.80	Validation
[114593-85-0]	Hydrocortisone succinamate	−4.59	461.60	1.43	Validation
[50-23-7]	Hydrocortisone	−5.52	362.50	1.61	Validation
[466-99-9]	Hydromorphone	−4.82	285.34	1.60	Training
[12041-98-4]	Hydroxypregnenolone	−3.22	332.50	3.71	Training
[68-96-2]	Hydroxyprogesterone	−3.22	330.50	3.17	Validation
[53-86-1]	Indomethacin	−3.67	357.80	4.27	Training
[119-65-3]	Isoquinoline	−1.78	129.20	2.08	Training
[57-42-1]	Meperidine	−2.43	247.40	2.72	Training
[67-56-1]	Methanol	−3.30	32.00	−0.77	Training
[99-76-3]	Methylhydroxybenzoate	−2.04	152.10	1.96	Training

[57-27-2]	Morphine	−5.03	285.30	0.89	Training
[22204-53-1]	Naproxen	−3.15	230.30	3.18	Training
[71-36-3]	*N*-Butanol	−2.52	74.14	0.88	Training
[112-30-1]	*N*-Decanol	−1.10	158.30	4.57	Training
[111-70-6]	*N*-Heptanol	−1.50	116.20	2.62	Training
[111-27-3]	*N*-Hexanol	−1.89	102.20	2.03	Validation
[54-11-5]	Nicotine	−1.72	162.30	1.17	Training
[55-63-0]	Nitroglycerine	−1.96	227.10	1.62	Validation
[1116-54-7]	*N*-Nitrosodiethanolamine	−5.22	134.13	−1.28	Training
[111-87-5]	*N*-Octanol	−1.28	130.20	3.00	Training
[143-08-8]	Nonanol	−1.22	144.30	3.77	Training
[71-41-0]	*N*-Pentanol	−2.22	88.20	1.51	Training
[71-23-8]	*N*-Propanol	−2.77	60.10	0.25	Training
[124-07-2]	Octanoic acid	−1.60	144.21	3.05	Training
[630-60-4]	Ouabain	−6.11	584.60	−2.00	Training
[109-52-4]	Pentanoic acid	−2.70	102.13	1.39	Validation
[50-06-6]	Phenobarbital	−3.35	232.20	1.47	Training
[108-95-2]	Phenol	−1.71	94.10	1.46	Training
[36322-90-4]	Piroxicam	−3.81	331.40	3.06	Training
[145-13-1]	Pregnenolone	−2.82	316.50	4.22	Training
[57-83-0]	Progesterone	−2.82	314.50	3.87	Training
[69-72-7]	Salicylic acid	−2.20	138.10	2.26	Training
[51-34-3]	Scopolamine	−4.30	303.40	0.98	Training
[100-42-5]	Styrene	−0.19	104.15	2.95	Training
[57-50-1]	Sucrose	−5.28	342.30	−3.07	Training
[56030-54-7]	Sufentanil	−2.26	386.60	3.95	Training
[58-22-0]	Testosterone	−3.40	288.40	3.32	Validation
[89-83-8]	Thymol	−1.28	150.20	3.30	Validation
[57-13-6]	Urea	−3.83	60.60	−2.11	Training
[7732-18-5]	Water	−3.30	18.00	−1.38	Training

Table S2. List of values estimated for molecular descriptors and skin permeability using quantitative structure-activity relationship developed in this study for compounds applied in model training and validation. Relevant information includes the value of molecular descriptors consisting of those for the distance/detour ring index of order 10 (D/Dr10), the sum of topological distances between O..Cl (T(O..Cl)), the Ghose-Crippen octanol-water partition coefficient (ALOGP), and the Ghose-Viswanadhan-Wendoloski antineoplastic-like index at 80% (Neoplastic-80) and the logarithmic value of skin permeability predicted by the model (predicted log *Kp*) for the 106 compounds included in the training and validation datasets.

**Chemical Name**	**Chemical Formula**	**MW**	**log K_{OW}**	**Observed log *Kp* (cm/h)**	**D/Dr10**	**T(O..Cl)**	**ALOGP**	**Neoplastic-80**	**Predicted log *Kp* (cm/h)**
1,1,1-Trichloroethane	$C_2H_3Cl_3$	133.40	2.49	−2.35	0	0	2.029	0	−1.71
2,4,6-Trichlorophenol	$C_6H_3Cl_3O$	197.50	3.69	−1.23	0	11	3.556	0	−1.21
2,4-Dichlorophenol	$C_6H_4Cl_2O$	163.00	3.06	−1.22	0	8	2.891	0	−1.51
2-Amino-4-nitrophenol	$C_6H_6N_2O_3$	154.10	1.26	−3.18	0	0	0.71	0	−2.61
2-Chlorophenol	C_6H_5ClO	128.60	2.15	−1.48	0	3	2.227	0	−1.72
o-Cresol	C_7H_8O	108.10	1.95	−1.80	0	0	2.049	0	−1.69
2-Ethoxyethanol	$C_4H_{10}O_2$	90.10	−0.32	−3.60	0	0	−0.14	0	−3.19
2-Nitro-*p*-phenylenediamine	$C_6H_7N_3O_2$	153.10	0.53	−3.30	0	0	0.231	0	−2.94
o-Phenylenediamine	$C_6H_8N_2$	108.10	0.15	−3.35	0	0	0.337	0	−2.86
2-Phenylethanol	$C_8H_{10}O$	122.20	1.36	−1.42	0	0	1.547	0	−2.04
3,4-Xylenol	$C_8H_{10}O$	122.10	2.30	−1.44	0	0	2.535	0	−1.36
m-Cresol	C_7H_8O	108.10	1.96	−1.82	0	0	2.049	0	−1.69
3-Nitrophenol	$C_6H_5NO_3$	139.10	2.00	−2.25	0	0	1.457	0	−2.10
4-Amino-2-nitrophenol	$C_6H_6N_2O_3$	154.10	0.96	−2.55	0	0	0.71	0	−2.61
4-Bromophenol	C_6H_5BrO	173.00	2.59	−1.44	0	0	2.311	0	−1.51
4-Chloro-*m*-phenylenediamine	$C_6H_7ClN_2$	142.60	0.85	−2.68	0	0	1.001	0	−2.41
4-Chlorophenol	C_6H_5ClO	128.60	2.39	−1.44	0	5	2.227	0	−1.82
p-Cresol	C_7H_8O	108.10	1.94	−1.76	0	0	2.049	0	−1.69
4-Ethylphenol	$C_8H_{10}O$	122.20	2.58	−1.46	0	0	2.505	0	−1.38
4-Nitrophenol	$C_6H_5NO_3$	139.10	1.91	−2.25	0	0	1.457	0	−2.10
p-Phenylenediamine	$C_6H_8N_2$	108.10	−0.30	−3.62	0	0	0.337	0	−2.86

Aldosterone	$C_{21}H_{28}O_5$	360.40	1.08	−5.52	140.399	0	1.118	1	−4.85
Amylobarbital	$C_{11}H_{18}N_2O_3$	226.30	2.07	−2.64	0	0	1.909	1	−3.36
Anisole	C_7H_8O	108.10	2.11	−1.60	0	0	1.814	0	−1.85
Atropine	$C_{17}H_{23}NO_3$	289.40	1.83	−5.07	0	0	1.721	1	−3.49
Barbital	$C_8H_{12}N_2O_3$	184.20	0.65	−3.95	0	0	0.745	1	−4.16
Benzaldehyde	C_7H_6O	106.10	1.48	−0.85	0	0	1.589	0	−2.01
Benzene	C_6H_6	78.10	2.13	−0.95	0	0	1.83	0	−1.84
Benzyl alcohol	C_7H_8O	108.10	1.10	−2.22	0	0	1.226	0	−2.26
b-Naphtol	$C_{10}H_8O$	144.20	2.70	−1.55	34.804	0	2.471	0	−1.64
Butobarbital	$C_{10}H_{16}N_2O_3$	212.40	1.73	−3.72	0	0	1.657	1	−3.53
Butyric acid	$C_4H_8O_2$	88.11	0.79	−3.00	0	0	0.893	0	−2.48
Chlorocresol	C_7H_7ClO	142.60	3.10	−1.26	0	5	2.713	0	−1.49
Chloroxylenol	C_8H_9ClO	156.60	3.27	−1.23	0	5	3.199	0	−1.15
Chlorpheniramine	$C_{16}H_{19}ClN_2$	274.80	3.38	−2.66	0	0	3.697	1	−2.14
Codeine	$C_{18}H_{21}NO_3$	299.40	1.19	−4.31	135.67	0	1.638	1	−4.46
Cortexolone	$C_{21}H_{30}O_4$	346.50	3.08	−4.13	133.416	0	2.386	1	−3.93
Cortexone	$C_{21}H_{30}O_3$	330.50	2.88	−3.35	126.325	0	3.122	1	−3.38
Corticosterone	$C_{21}H_{30}O_4$	346.50	1.94	−4.22	132.084	0	1.952	1	−4.22
Cortisone	$C_{21}H_{28}O_5$	360.50	1.47	−5.00	139.176	0	1.034	1	−4.89
Diclofenac	$C_{14}H_{11}Cl_2NO_2$	318.00	4.51	−3.45	0	32	4.348	1	−3.28
Diethylcarbamazine	$C_{10}H_{21}N_3O$	199.30	0.37	−3.89	0	0	0.436	1	−4.37
Digitoxin	$C_{41}H_{64}O_{13}$	764.90	1.85	−4.89	427.064	0	3.101	0	−3.85
Dihydromorphine	$C_{17}H_{21}NO_3$	287.36	0.93	−4.82	125.421	0	1.696	1	−4.35
Ephedrine	$C_{10}H_{15}NO$	165.20	1.13	−2.22	0	0	1.235	0	−2.25
Estradiol	$C_{18}H_{24}O_2$	272.40	4.01	−2.28	95.835	0	3.813	1	−2.70
Estriol	$C_{18}H_{24}O_3$	288.40	2.45	−4.40	103.496	0	2.846	1	−3.42
Estrone	$C_{18}H_{22}O_2$	270.40	3.13	−2.44	95.835	0	3.773	1	−2.73
Ethanol	C_2H_6O	46.10	−0.31	−3.00	0	0	−0.009	0	−3.10

Ethyl ether	$C_4H_{10}O$	74.10	0.89	−2.80	0	0	0.748	0	−2.58
Etorphine	$C_{25}H_{33}NO_4$	411.50	2.79	−2.44	154.042	0	2.928	0	−2.13
Fentanyl	$C_{22}H_{28}N_2O$	336.50	4.05	−2.25	0	0	3.839	1	−2.04
Fluocinonide	$C_{26}H_{32}F_2O_7$	494.60	3.19	−2.77	185.404	0	1.308	0	−3.45
Heptanoic acid	$C_7H_{14}O_2$	130.19	2.42	−1.70	0	0	2.262	0	−1.55
Heptanol	$C_7H_{16}O$	116.20	2.31	−1.49	0	0	2.339	0	−1.49
Hexanoic acid	$C_6H_{12}O_2$	116.16	1.92	−1.85	0	0	1.806	0	−1.86
Hydrocortisone hemipimelate	$C_{28}H_{40}O_8$	503.60	3.26	−2.75	247.224	0	2.784	0	−2.85
Hydrocortisone hemisuccinate	$C_{25}H_{34}O_8$	462.50	1.89	−3.20	210.811	0	1.416	0	−3.54
Hydrocortisone hexanoate	$C_{27}H_{40}O_6$	460.60	4.48	−1.75	211.188	0	3.631	0	−2.03
Hydrocortisone hydroxyhexanoate	$C_{27}H_{40}O_7$	476.60	2.79	−3.04	222.987	0	2.406	0	−2.95
Hydrocortisone methylpimelate	$C_{29}H_{42}O_8$	518.60	3.70	−2.27	259.638	0	3.035	0	−2.77
Hydrocortisone methylsuccinate	$C_{26}H_{36}O_8$	476.60	2.60	−3.68	222.265	0	1.667	0	−3.45
Hydrocortisone *N*,*N*-dimethylsuccinamate	$C_{27}H_{39}NO_7$	489.60	2.03	−4.17	233.718	0	1.225	0	−3.83
Hydrocortisone octanoate	$C_{29}H_{44}O_6$	488.70	5.49	−1.21	235.106	0	4.544	0	−1.57
Hydrocortisone pimelamate	$C_{28}H_{41}NO_7$	503.60	2.31	−3.05	247.224	0	2.182	0	−3.27
Hydrocortisone propionate	$C_{24}H_{34}O_6$	418.50	2.80	−2.47	177.992	0	2.263	0	−2.74
Hydrocortisone succinamate	$C_{25}H_{35}NO_7$	461.60	1.43	−4.59	210.811	0	0.814	0	−3.96
Hydrocortisone	$C_{21}H_{30}O_5$	362.50	1.61	−5.52	139.176	0	1.217	1	−4.77
Hydromorphone	$C_{17}H_{19}NO_3$	285.34	1.60	−4.82	125.421	0	1.657	1	−4.38
Hydroxypregnenolone	$C_{21}H_{32}O_3$	332.50	3.71	−3.22	124.851	0	2.577	1	−3.74
Hydroxyprogesterone	$C_{21}H_{30}O_3$	330.50	3.17	−3.22	124.863	0	2.845	1	−3.56
Indomethacin	$C_{19}H_{16}ClNO_4$	357.80	4.27	−3.67	0	39	4.211	1	−3.72
Isoquinoline	C_9H_7N	129.20	2.08	−1.78	29.717	0	1.588	0	−2.21
Meperidine	$C_{15}H_{21}NO_2$	247.40	2.72	−2.43	0	0	2.446	1	−2.99

Methanol	CH_4O	32.00	−0.77	−3.30	0	0	−0.358	0	−3.34
Methylhydroxybenzoate	$C_8H_8O_3$	152.10	1.96	−2.04	0	0	1.418	0	−2.12
Morphine	$C_{17}H_{19}NO_3$	285.30	0.89	−5.03	125.421	0	0.381	1	−5.25
Naproxen	$C_{14}H_{14}O_3$	230.30	3.18	−3.15	68.623	0	2.824	1	−3.20
N-Butanol	$C_4H_{10}O$	74.14	0.88	−2.52	0	0	0.971	0	−2.43
N-Decanol	$C_{10}H_{22}O$	158.30	4.57	−1.10	0	0	3.708	0	−0.56
N-Heptanol	$C_7H_{16}O$	116.20	2.62	−1.50	0	0	2.339	0	−1.49
N-Hexanol	$C_6H_{14}O$	102.20	2.03	−1.89	0	0	1.883	0	−1.81
Nicotine	$C_{10}H_{14}N_2$	162.30	1.17	−1.72	0	0	1.243	0	−2.24
Nitroglycerine	$C_3H_5N_3O_9$	227.10	1.62	−1.96	0	0	0.078	0	−3.04
N-Nitrosodiethanolamine	$C_4H_{10}N_2O_3$	134.13	−1.28	−5.22	0	0	−0.988	0	−3.77
N-Octanol	$C_8H_{18}O$	130.20	3.00	−1.28	0	0	2.796	0	−1.18
Nonanol	$C_9H_{20}O$	144.30	3.77	−1.22	0	0	3.252	0	−0.87
N-Pentanol	$C_5H_{12}O$	88.20	1.51	−2.22	0	0	1.427	0	−2.12
N-Propanol	C_3H_8O	60.10	0.25	−2.77	0	0	0.515	0	−2.74
Octanoic acid	$C_8H_{16}O_2$	144.21	3.05	−1.60	0	0	2.718	0	−1.24
Ouabain	$C_{29}H_{44}O_{12}$	584.60	−2.00	−6.11	252.634	0	−2.171	0	−6.28
Pentanoic acid	$C_5H_{10}O_2$	102.13	1.39	−2.70	0	0	1.349	0	−2.17
Phenobarbital	$C_{12}H_{12}N_2O_3$	232.20	1.47	−3.35	0	0	1.321	1	−3.76
Phenol	C_6H_6O	94.10	1.46	−1.71	0	0	1.563	0	−2.03
Piroxicam	$C_{15}H_{13}N_3O_4S$	331.40	3.06	−3.81	97.236	0	1	1	−4.64
Pregnenolone	$C_{21}H_{32}O_2$	316.50	4.22	−2.82	117.771	0	3.485	1	−3.07
Progesterone	$C_{21}H_{30}O_2$	314.50	3.87	−2.82	117.771	0	3.58	1	−3.01
Salicylic acid	$C_7H_6O_3$	138.10	2.26	−2.20	0	0	1.167	0	−2.30
Scopolamine	$C_{17}H_{21}NO_4$	303.40	0.98	−4.30	0	0	0.824	1	−4.10
Styrene	C_8H_8	104.15	2.95	−0.19	0	0	2.382	0	−1.47
Sucrose	$C_{12}H_{22}O_{11}$	342.30	−3.07	−5.28	0	0	−4.311	0	−6.04
Sufentanil	$C_{22}H_{30}N_2O_2S$	386.60	3.95	−2.26	0	0	3.231	1	−2.46

Testosterone	$C_{19}H_{28}O_2$	288.40	3.32	−3.40	102.038	0	3.333	1	−3.07
Thymol	$C_{10}H_{14}O$	150.20	3.30	−1.28	0	0	3.243	0	−0.88
Urea	CH_4N_2O	60.60	−2.11	−3.83	0	0	−1.042	0	−3.81
Water	H_2O	18.00	−1.38	−3.30	0	0	−0.564	0	−3.48
